# Enhanced health event detection and influenza surveillance using a joint Veterans Affairs and Department of Defense biosurveillance application

**DOI:** 10.1186/1472-6947-11-56

**Published:** 2011-09-19

**Authors:** Cynthia A Lucero, Gina Oda, Kenneth Cox, Frank Maldonado, Joseph Lombardo, Richard Wojcik, Mark Holodniy

**Affiliations:** 1 Department of Veterans Affairs, Office of Public Health, Office of Public Health Surveillance and Research, 3801 Miranda Ave (132), Palo Alto, CA 94304, USA; 2 Medical Informatics and Special Projects, Armed Forces Health Surveillance Center, 2900 Linden Lane, Suite 200, Silver Springs, MD 20910, USA; 3 Associate Chief of Staff for Clinical Affairs, Department of Veterans Affairs, Captain James A. Lovell Federal Health Care Center, 3001 Green Bay Rd. North Chicago, IL 60064, USA; 4 Johns Hopkins University Applied Physics Laboratory, ESSENCE Group, 11100 Johns Hopkins Road, Laurel, MD 20723, USA

## Abstract

**Background:**

The establishment of robust biosurveillance capabilities is an important component of the U.S. strategy for identifying disease outbreaks, environmental exposures and bioterrorism events. Currently, U.S. Departments of Defense (DoD) and Veterans Affairs (VA) perform biosurveillance independently. This article describes a joint VA/DoD biosurveillance project at North Chicago-VA Medical Center (NC-VAMC). The Naval Health Clinics-Great Lakes facility physically merged with NC-VAMC beginning in 2006 with the full merger completed in October 2010 at which time all DoD care and medical personnel had relocated to the expanded and remodeled NC-VAMC campus and the combined facility was renamed the Lovell Federal Health Care Center (FHCC). The goal of this study was to evaluate disease surveillance using a biosurveillance application which combined data from both populations.

**Methods:**

A retrospective analysis of NC-VAMC/Lovell FHCC and other Chicago-area VAMC data was performed using the ESSENCE biosurveillance system, including one infectious disease outbreak (Salmonella/Taste of Chicago-July 2007) and one weather event (Heat Wave-July 2006). Influenza-like-illness (ILI) data from these same facilities was compared with CDC/Illinois Sentinel Provider and Cook County ESSENCE data for 2007-2008.

**Results:**

Following consolidation of VA and DoD facilities in North Chicago, median number of visits more than doubled, median patient age dropped and proportion of females rose significantly in comparison with the pre-merger NC-VAMC facility. A high-level gastrointestinal alert was detected in July 2007, but only low-level alerts at other Chicago-area VAMCs. Heat-injury alerts were triggered for the merged facility in June 2006, but not at the other facilities. There was also limited evidence in these events that surveillance of the combined population provided utility above and beyond the VA-only and DoD-only components. Recorded ILI activity for NC-VAMC/Lovell FHCC was more pronounced in the DoD component, likely due to pediatric data in this population. NC-VAMC/Lovell FHCC had two weeks of ILI activity exceeding both the Illinois State and East North Central Regional baselines, whereas Hines VAMC had one and Jesse Brown VAMC had zero.

**Conclusions:**

Biosurveillance in a joint VA/DoD facility showed potential utility as a tool to improve surveillance and situational awareness in an area with Veteran, active duty and beneficiary populations. Based in part on the results of this pilot demonstration, both agencies have agreed to support the creation of a combined VA/DoD ESSENCE biosurveillance system which is now under development.

## Background

The United States faces many potential threats to human health, including disease outbreaks, accidental environmental exposures, and acts of terrorism. Effective biosurveillance at a government agency level is dependent upon representative and timely data collection, proper analysis and interpretation of data at the local and regional levels, and incorporation of the national perspective and visualization techniques to provide a broad, accurate picture across regions or jurisdictions. Achieving these steps is critical in order to obtain early warning of health risks, early detection of health events and overall situational awareness of disease or potential bioterrorist activity.

Developing robust biosurveillance capabilities within government agencies and having the ability to share information across agencies has become increasingly important in recent times [1]. Linkage of federal agency biosurveillance capabilities and health data exchange is an important component of multiple laws, initiatives and directives including Public Law 110-53 (Implementing Recommendations of the 9/11 Commission Act of 2007) [2], the National Biosurveillance Integration Center (NBIC) code [3], Homeland Security Presidential Directive (HSPD)-10 (Biodefense for the 21^st ^Century) [4], HPSD-21 (Public Health and Medical Preparedness) [5], Public Law 109-417 (Pandemic All-Hazards and Preparedness Act) [6], the Federal Health Architecture initiative [7], and the National Biosurveillance Strategy for Human Health [8]. The Departments of Defense (DoD) and Veterans Affairs (VA) are among the largest healthcare systems providing care in the United States. In 2010, the VA healthcare system had 8.3 million Veterans enrolled and over 75 million out-patient visits and 679,600 inpatient admissions [9]. In 2010, the Military Health System (TRICARE) had 9.6 million eligible beneficiaries and approximately 42 million direct care out-patient visits and 265,000 inpatient admissions [10]. Persons who served in the active military and were discharged or released under conditions other than dishonorable may qualify for VA healthcare benefits. The VA and DoD healthcare systems operate independently and maintain separate electronic health record (EHR) systems. In 2004 there was a recognized need to exchange information between the two EHRs in order to provide a more seamless transition of care from DoD to VA. In response, the Federal Health and Bidirectional Health Information Exchange (FHIE/BHIE) interagency program was launched to permit secure, real-time exchange of medical record data between VA and DoD. However, the information exchange is currently incomplete and there is no comprehensive system in place that allows for streamlined transition of health care records between the two agencies. In 2009, President Obama, VA Secretary Shinseki and DoD Secretary Gates announced that DoD and VA had taken the first steps in creating a unified Virtual Lifetime Electronic Record (VLER) [11]. The program is currently in a pilot phase, testing exchange of information over the Nationwide Health Information Network (NHIN). In the future, this system will provide connectivity that has never before been accomplished and will greatly improve access to health information within the federal government as well as private sector partners nationwide, providing a continuous health record from time of enlistment to death.

Both Departments are committed to developing and maintaining state-of-the-art biosurveillance systems for monitoring disease activity and emerging health threats. Despite the fact that many DoD and VA facilities exist in common geographic areas, biosurveillance is currently being performed independently by each Department using a system developed by the Johns Hopkins University Applied Physics Laboratory (JHUAPL) and Walter Reed Army Institute of Research known as ESSENCE (Electronic Surveillance System for the Early Notification of Community-Based Epidemics) [12-14]. ESSENCE is also the primary biosurveillance system in place at many state and local health departments, however these systems do not contain any VA or DoD hospital data in their applications [15-18]. The DoD and VA ESSENCE systems utilize complex algorithms to evaluate *International Classification of Diseases, 9^th ^Revision (ICD-9) *diagnosis codes from outpatient and emergency department visits. Codes are clustered into broad syndrome groups which are designed to capture both naturally occurring disease outbreaks and potential bioterrorist events. Although the two systems provide very similar functionality, they are completely independent and there is no formal sharing of biosurveillance data between systems or across agencies.

VA and DoD recognize that performing joint health surveillance could provide the agencies' public health personnel with additional indicators that may not be detectable in their respective individual surveillance populations. As an example, influenza outbreaks are often first seen in the pediatric population and may spread more rapidly among the very young given the extent of their social interactions. Therefore, early influenza activity and trends may be missed by VA surveillance which has little to no pediatric data. On the other hand, older patients and those with multiple co-morbid conditions often suffer much higher morbidity and mortality due to influenza. As a result, the severity of an influenza season or pandemic may be underestimated by the DoD system, which is overrepresented by younger and otherwise healthy individuals. In addition, combining surveillance populations provides a larger statistical sample of total population in the region for earlier recognition of disease events and monitoring the spread of a communicable disease through a community. Prior evaluations of biosurveillance systems with multiple data streams found that data from different sources was useful for detecting and monitoring disease outbreaks and provide a substantial alerting-timeliness advantage [19-21]. ESSENCE has previously been linked with disparate systems for real-time data exchange and communication [22]. In that project, data sharing was simplified though a service-oriented architecture and the accurate and timely information exchange that was achieved displayed potential to enhance effective collaboration and improve patient care. A joint surveillance approach would likely also provide additional power for the detection of rare disease events, emerging health threats, and bioterrorist attacks. The clinical application of an enhanced, joint surveillance system would be beneficial to healthcare providers and public health practitioners in both Departments.

Although the VA and DoD ESSENCE systems are not currently integrated, an opportunity to evaluate the benefits of combined surveillance exists in North Chicago, IL where VA and DoD have partnered to create the first fully integrated federal healthcare facility. The Great Lakes Naval Training Center is the home to the U.S. Navy's only Recruit Training Command and is the central processing location for all Naval recruits. The majority of trainees are in the 18-25 year age group. Starting in June 2006, the Naval Health Clinics-Great Lakes inpatient, surgical, emergency and some limited outpatient services for active duty personnel and their dependents were relocated to North Chicago Veterans Affairs Medical Center (NC-VAMC). This consolidation includes a project to increase interoperability between VA and DoD electronic health records by allowing bidirectional flow of data. Full integration of all inpatient and outpatient services occurred October 1, 2010 when all DoD care and medical personnel had relocated to the expanded and remodeled NC-VAMC campus and at that time the facility was officially renamed Captain James A. Lovell Federal Health Care Center (Lovell FHCC). Both agencies have agreed to maintain and support each others' main mission, highest quality patient care for Veterans and operational readiness of military personnel. The merger of these VA and DoD facilities provided an opportunity to evaluate data in a combined VA/DoD patient population without the establishment of a formal biosurveillance sharing agreement or data exchange. Biosurveillance data from active duty military personnel and their dependents that have been seen and treated at NC-VAMC/Lovell FHCC starting June 1, 2006, is available in the VA ESSENCE system for analysis.

The objectives of this study were: 1) Describe changes in facility and patient demographics resulting from the VA/DoD integration in North Chicago, 2) Determine if health event detection and influenza surveillance in the joint facility was enhanced compared with two other VA-only facilities in the Chicago-metro area (Jesse Brown VAMC and Hines VAMC), 3) Examine the combined surveillance data in relation to the VA-only and DoD-only components within ESSENCE.

## Methods

### Overview

The study was a retrospective analysis of VA ESSENCE surveillance data for the three Chicago-area VAMCs (NC-VAMC/Lovell FHCC, Jesse Brown VAMC and Hines VAMC). The NC-VAMC/Lovell FHCC is located in Lake County, IL, 33 miles north of downtown Chicago and provides care for active duty military, beneficiaries and Veterans throughout Northern Illinois and Southern Wisconsin. The Jesse Brown VAMC is located in adjacent Cook County, IL and provides care to enrolled Veterans in the City of Chicago; Cook County, IL; and in six counties in northwestern Indiana. The Hines VAMC is located 12 miles west of downtown Chicago also in Cook County, IL and provides care to Veterans primarily from Cook, DuPage, La Salle and Will counties. The Stanford University institutional review board approved this study. Analysis was conducted using VA ESSENCE Version 1.4. The system collects information from all VA Medical Centers and community based outpatient clinics (CBOCs) in all 50 states, plus Guam, American Samoa, Puerto Rico, U.S. Virgin Islands and the Philippines. VA ESSENCE pulls ICD-9 diagnosis codes and demographic data from all outpatient and emergency department visits. A limited number of inpatient visits and consultations are also captured. Visits are grouped into a VA ESSENCE syndrome category if one or more of the assigned ICD-9 code(s) for the visit matches a defined code for the syndrome. Syndrome categories include: Botulism-like, Febrile Disease, Fever, Gastrointestinal (GI), Hemorrhagic Illness, Influenza-like Illness (ILI), Miscellaneous/Asthma, Neurological, Rash, Respiratory and Shock/Coma. ICD-9 codes can be analyzed individually or at the syndrome level.

Data are analyzed by the system's aberrancy-detection algorithms to identify when observed counts are significantly above predicted values. The alerting algorithm methodology for ESSENCE syndromic surveillance data has been described previously [23,24]. VA ESSENCE determines an expected count by employing regression modeling based on historical data, day-of-the week effects, seasonal trends and effects due to other factors. After determining the expected count, the system applies statistics and runs significance tests for each syndrome or ICD-9 code to determine whether the observed counts are reasonably close to what is expected from the model predictions. When the test for reasonable agreement fails, VA ESSENCE produces flags (red or yellow alerts) to indicate a count, which is significantly above the predicted level. Alerts are designed to warn the system user of a possible outbreak or cluster. The tests for reasonable agreement employ confidence intervals (CIs). When the observed count falls between the 95% and 99% CI a low-level (yellow) alert is generated. If the count exceeds the 99% CI, a high-level (red) alert is triggered.

### Study Design

We compared aggregate patient and facility demographic data in VA ESSENCE prior to (August 2005-May 2006) and after (June 2006-March 2009) integration began at the VA and DoD North Chicago facilities. Data elements evaluated included: total number of visits captured per month, patient age and gender, syndrome groups and ICD-9 diagnosis codes. Data were exported from VA ESSENCE to Microsoft Excel for analysis. For visits with an ICD-9 code appearing in multiple syndrome categories, as well as those visits assigned multiple different ICD-9 codes, and in cases where the patient had more than one outpatient visit on a single date, duplicates were identified and removed in Microsoft Excel to ensure that each visit was counted only once in the analysis. Because the live DoD ESSENCE system only maintains the most recent 18 months of data, DoD data from the 2005-2006 pre-integration time period was not readily available through the active DoD ESSENCE application. However, archived DoD ESSENCE emergency department (ED) data was obtained from electronic storage for comparison, as it was this department which initially transferred to the VA facility at the start of the merger. Descriptive statistics and univariate analysis for this study were performed using SAS 9.1 (SAS Institute, Cary, NC).

We searched the Chicago Department of Public Health online archives for disease outbreaks and major health events occurring between June 2006 and March 2009. The archive consisted primarily of press releases and health warnings issued by the health department. We attempted to identify events that were large in magnitude as well as ones with the potential to affect the entire metro-Chicago area in order to maximize the chances of activity within the Chicago-area VA system. The majority of events were not suitable for this analysis as they involved only a small number of cases or were limited to a single non-VA hospital or other facility. However, we identified and selected one infectious disease outbreak (Salmonella, Taste of Chicago Festival, July 2007) and one weather-related health event (Heat Wave, multiple days, July 2006) in the Chicago-metro area for evaluation. These reports were the two identified which represented large regional events that were most likely to have affected the cities of Chicago and North Chicago. The Taste of Chicago is one of the world's largest annual food festivals, beginning the Friday before Independence Day and ending the Sunday after. During the 2007 event (June 29-July 8, 2007), a Salmonella outbreak occurred in which approximately 800 people became ill and nearly 40 were hospitalized. The outbreak was eventually traced to hummus served by a single vendor [25,26]. The second event was the 2006 North America heat wave, which spread through most of the United States and Canada, producing extreme temperatures, which led to the death of at least 225 people. Chicago was among the U.S. cities affected during July and August of 2006. Extreme temperature advisories were issued by the Chicago City Health Department on July 14 and 31, 2006 [27,28]. National Weather Service climate summary for Chicago 2006, reported that the first wave occurred from July 13-17^th ^with the warmest day on July 15^th ^when the high reached 97 degrees. The second wave was approximately July 29-August 4^th ^with the warmest days on July 31^st ^and August 1^st ^with high temperatures of 99 degrees both days [29]. Neither the GI nor the heat wave event was reviewed in our systems prior to selection for analysis in this project. For this analysis, we reviewed VA ESSENCE gastrointestinal syndrome and heat-injury data (ICD-9: 992) during these two time periods at each of the three Chicago VAMCs. Regression/Exponentially Weighted Moving Average (EWMA) was used as the VA ESSENCE alerting algorithm.

Finally, we used available VA ESSENCE data to determine the weekly percentage of visits for ILI at each Chicago-area facility for the 2007-2008 influenza season and compared it with the Centers for Disease Control and Prevention (CDC) regional baseline for the East North Central region (Illinois, Indiana, Michigan, Ohio and Wisconsin) and the Illinois State baseline. We then plotted VA ESSENCE ILI data against the CDC/Illinois Sentinel Provider Network (ILINet) and Chicago's Cook County ESSENCE emergency department visits for ILI during the same influenza season. Data for the CDC/Illinois Sentinel Provider Network was obtained from the Illinois Department of Public Health Influenza Surveillance 2007-2008 Flu Activity Report [30]. Providers report number of patients seen and the number with ILI, defined as fever ≥ 100°F and a cough and/or sore throat. Data from the Cook County ESSENCE system was obtained from the ESSENCE Surveillance Data for Influenza-Like Illness from the Cook County Department of Public Health Sentinel Influenza Surveillance Report [31]. Twenty-five hospital EDs in the county participate in Cook County ESSENCE, however there is no VA or DoD data in their system. The Cook County ESSENCE ILI syndrome is defined as a symptom complex of fever and cough or sore throat. ILI data from the DoD ESSENCE system has previously been found to have a very strong correlation with CDC ILINet data [32]. For the VA ESSENCE system we included visits with an ICD-9 diagnosis code of influenza, fever, cough or sore throat to approximate the ILI definition used by ILINet and Cook County ESSENCE. We also limited the analysis to VA ED, urgent care and primary care clinics in order to more closely match the outpatient settings contained in these other systems.

## Results

**Figure 1:**
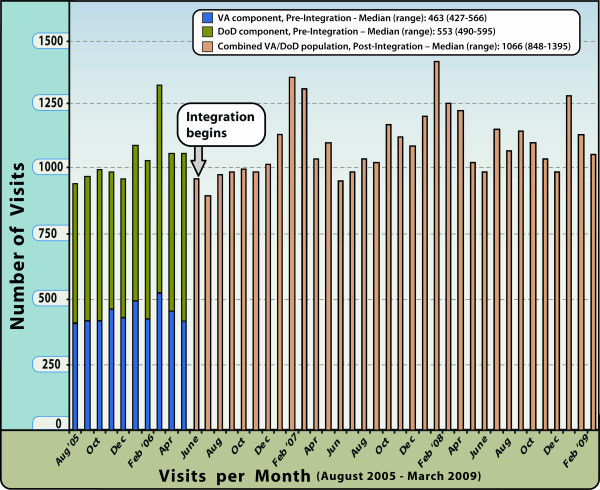
**Number of visits per month**. Display of the number of visits recorded per month at North Chicago VAMC/Lovell FHCC, Pre- and Post-Integration, August 2005-March 2009, ESSENCE Biosurveillance System.

**Figure 2:**
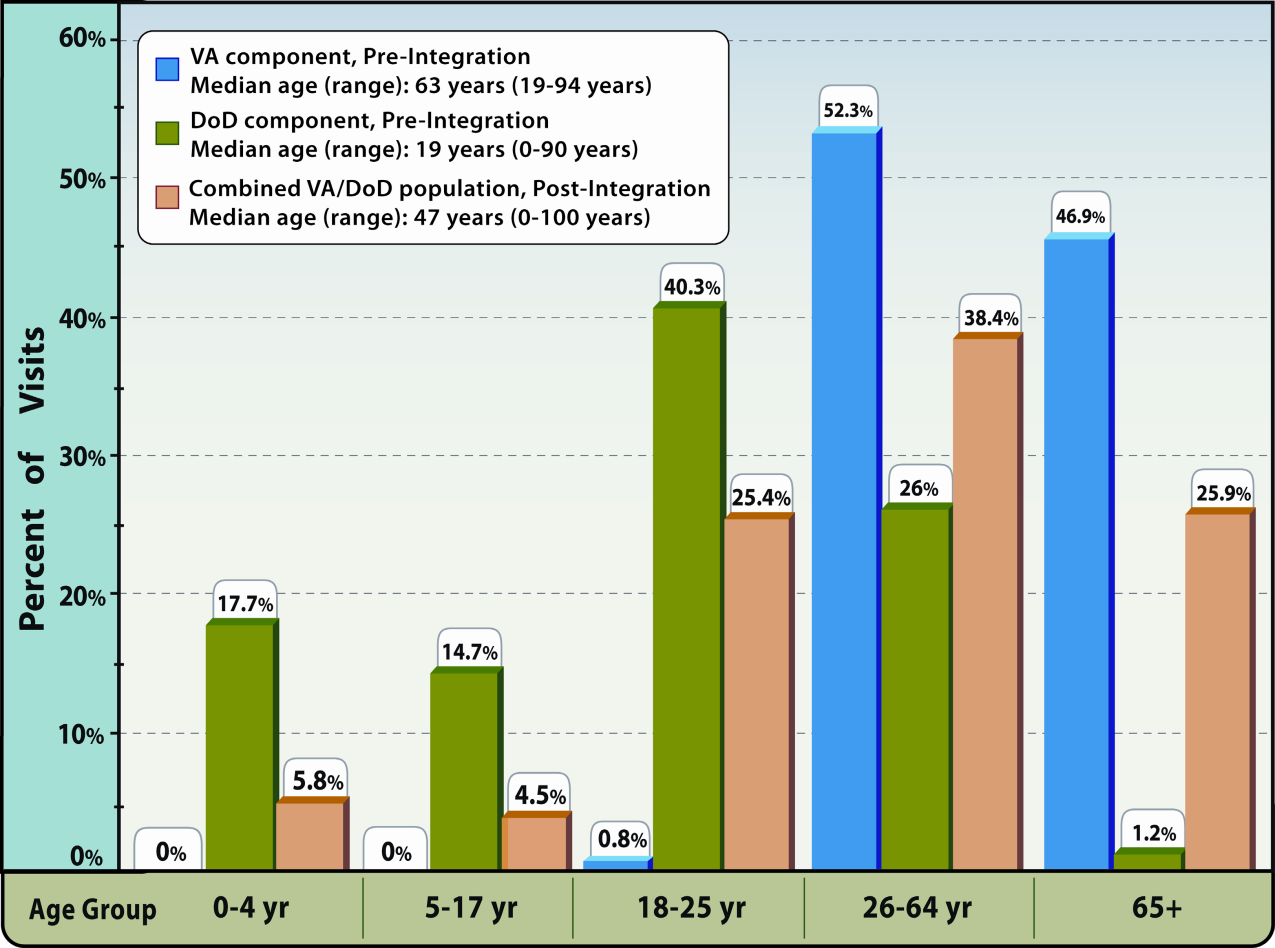
**Percentage of visits by age-group**. Display of the percentage of visits recorded for each age-group, North Chicago VAMC/Lovell FHCC, Pre- and Post-Integration, ESSENCE Biosurveillance System.

Since the beginning of the VA/DoD integration in North Chicago, the median number of visits captured by the system increased markedly from 463 per month to 1,066 per month (Figure 1, p < 0.01). The median number of DoD ED visits during the pre-integration period was 553 per month and dropped to zero beginning in June 2006 when these services were moved to NC-VAMC/Lovell FHCC. The median patient age for NC-VAMC/Lovell FHCC ESSENCE visits prior to June 2006 was 63 years, compared with 47 years after June 2006 (Figure 2, p < 0.01). For comparison, the median patient age for DoD ED visits in the pre-integration time period was 19 years. The most notable change at NC-VAMC/Lovell FHCC was the increase in the 18-25 year age-group as well as the addition of pediatric age-groups (Figure 2). The proportion of female patient visits at NC-VAMC/Lovell FHCC also rose from 11% to 25% (p < 0.01). For comparison, females accounted for 45% of DoD ED visits in the pre-integration period.

**Figure 3:**
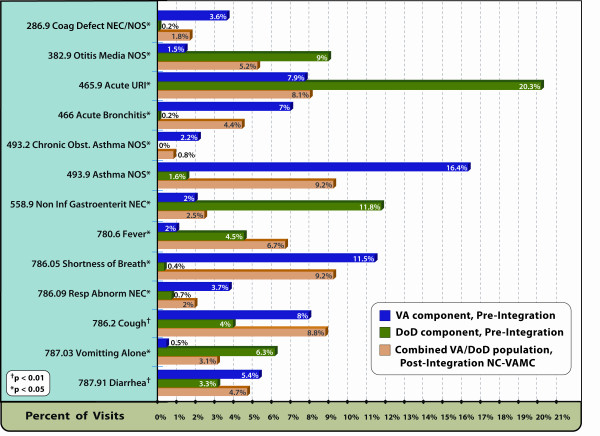
**Most frequently utilized ICD-9 diagnosis codes**. Display of the most frequently recorded ICD-9 diagnosis codes for the ESSENCE Biosurveillance syndrome categories, North Chicago VAMC/Lovell FHCC, Pre- and Post-Integration.

Significant changes were noted in the proportion of visits related to specific ICD-9 diagnosis codes at NC-VAMC/Lovell FHCC post-integration. Among the most frequently utilized diagnosis codes, a more than 6-fold increase was seen in the percentage of visits with the Fever code (ICD-9, 780.6) and Vomiting Alone (ICD-9 787.03) and a 3-fold increase in the Otitis Media NOS code (ICD-9, 382.9). Codes which declined post-integration included: Coagulation Defect NEC/NOS (ICD-9, 286.9), Acute Bronchitis (ICD-9, 466), Chronic Obstructive Asthma NOS (ICD-9, 493.2), Asthma NOS (ICD-9, 493.9), Shortness of Breath (ICD-9, 786.05), Respiratory Abnormality NEC (ICD-9, 786.09) and Diarrhea (ICD-9, 787.91) (Figure 3).

**Figure 4:**
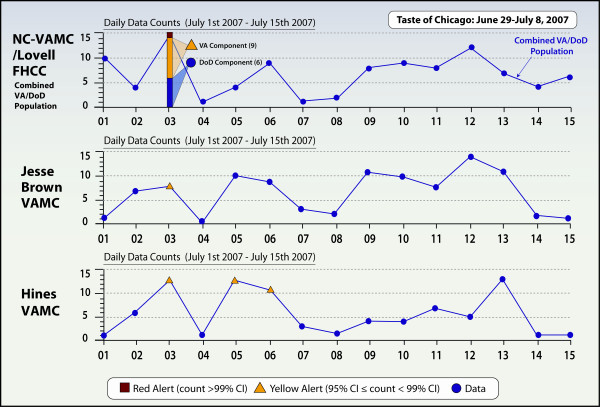
**Gastrointestinal (GI) syndrome counts**. Display of GI syndrome counts for three Chicago-area VA Medical Centers during the Taste of Chicago Salmonella outbreak, July 2007, ESSENCE Biosurveillance System.

Gastrointestinal syndrome counts for July 2007 during the Salmonella Taste of Chicago outbreak period revealed a single high-level alert in NC-VAMC/Lovell FHCC (15 visits on July 3, 2007). One low-level alert was found at Jesse Brown VAMC and 3 low-level alerts in Hines VAMC but no high-level alerts (Figure 4). A review of the patient eligibilities for the 15 visits at NC-VAMC/Lovell FHCC on July 3, 2007 found that 6 were DoD/TRICARE, 8 were Veterans and 1 patient was both a Veteran and VA employee. Five of the 15 were in the 18-25 year age-group. There was one pediatric patient (< 18 years) and 4 elderly patients (> 65 years). Additional ESSENCE analysis, which separated patient visits into the VA and DoD components, found that the DoD visits alone would not have fired an alert and the VA visits alone would have fired a low-level GI syndrome alert. Only the combined VA and DoD visits fired a high-level GI syndrome alert on that date.

**Figure 5:**
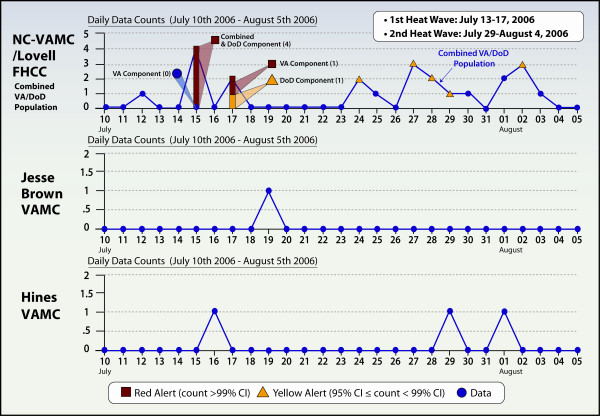
**Heat injury ICD-9 diagnosis codes**. Display of heat injury ICD-9 diagnosis codes (992) for three Chicago-area VA Medical Centers, July 10-August 5, 2006, ESSENCE Biosurveillance System.

Heat injury ICD-9 codes during the Heat Wave of 2006 identified 2 high-level alerts (4 visits on July 15, 2006 and 2 visits on July 17, 2006) and 5 low-level alerts in NC-VAMC/Lovell FHCC but no alerts for either Jesse Brown VAMC or Hines VAMC (Figure 5). A review of patient eligibilities for the 21 visits at NC-VAMC/Lovell FHCC with a heat injury (ICD-9: 992) code identified by VA ESSENCE between July 10-August 5, 2006 revealed that 15 were DoD/TRICARE, 3 were Veterans, 2 were humanitarian emergencies and 1 was a VA employee. Twelve of the 21 were in the 18-25 year age-group. There was only one pediatric patient (< 18 years) and no elderly patients (≥ 65 years). Additional VA ESSENCE analysis was performed which separated the patient populations for the two high-level alert dates. We found that for July 15, 2006 all four visits were DoD patients and therefore only the DoD component would have fired an alert. For July 17, 2006, one visit was a VA employee and one was a DoD patient. On this date, the single VA visit would have generated a high-level alert but the DoD visit would have generated a low-level alert. This is due to the fact that the expected count for the heat injury ICD-9 codes is slightly higher in the DoD population.

**Figure 6:**
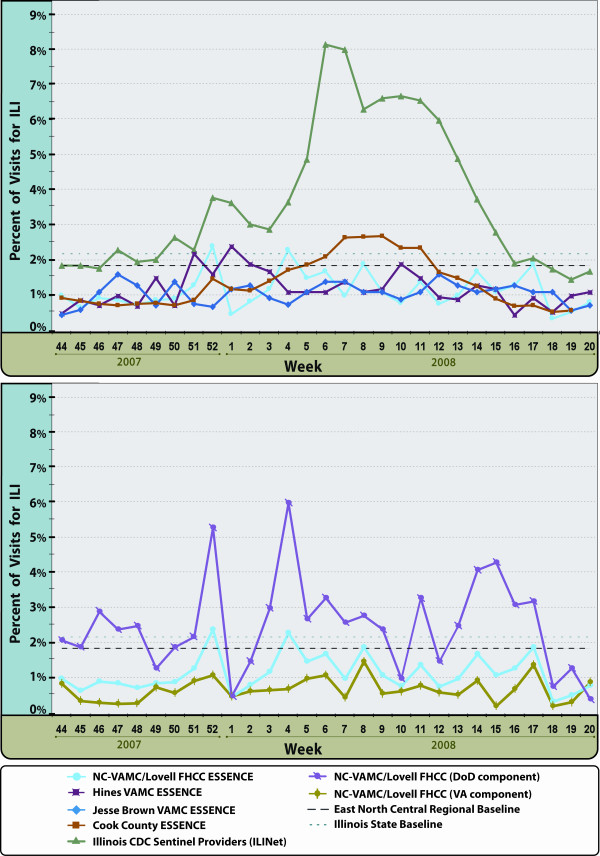
**Visits for Influenza-like Illness (ILI) by CDC Week**. Display of the percentage of visits with ILI during the 2007-2008 Influenza Season at Chicago-area VA Medical Centers in the VA ESSENCE Biosurveillance System compared with Cook County ESSENCE System and CDC's Illinois Sentinel Provider Network (ILINet) (Upper panel); and comparison of the combined VA/DoD population at North Chicago VAMC/Lovell FHCC with the VA and DoD patient components (Lower panel).

We reviewed ILI trends for the 2007-2008 influenza season. NC-VAMC/Lovell FHCC had two weeks of activity (Weeks 52 and 4) which exceeded both the U.S. East North Central regional baseline (1.9%) and the Illinois state baseline (2.2%), Hines VAMC had one (Week 1) and Jesse Brown had none. Overall, the peaks were less pronounced than those recorded by the Illinois Sentinel Providers (ILINet) and the Cook County ESSENCE system (Figure 6, upper panel). A closer examination of NC-VAMC/Lovell FHCC ILI trends showed more obvious peaks in the DoD component when compared to the VA component (Figure 6, lower panel).

## Discussion

The purpose of our evaluation was to investigate the potential advantages of a consolidated biosurveillance application combining the distinct and unique patient populations of our two agencies. This study represents the first example of merging and analysis of biosurveillance data between VA and DoD. Our study, which was conducted in a single integrated VA/DoD facility in North Chicago, highlights the improvements achieved in sample size, along with age and gender diversity in a combined VA/DoD patient pool. Although these changes were an expected outcome of the facility merger, it represents a unique patient mix, which does not exist in any other VA facility and has not been described previously. Median patient age in this facility fell by more than 15 years following the merger. Prior to integration, there were no visits in the pediatric age groups in the VA data, however post-integration they rose to more than 10% of the visits. The proportion of visits in the 65 and over age group fell from 47% to 26%. As a comparison, the U.S. Census Bureau ACS reported that 24% of the North Chicago population was under 18 years of age and just 6% was age 65 years and over [33]. Although the facility is not necessarily a representative sample of the community population, the patient mix in the merged facility now more closely resembles the population characteristics of North Chicago.

The analysis also revealed potential improvements in temporal alerting and flu trending demonstrated by the review of alerts generated for two large public health events and seasonal ILI trends when compared with two Chicago-area VA-only medical centers which do not include any pediatric data. For syndromes such as ILI where one population routinely demonstrates a higher concentration of disease or earlier peak in disease frequency, our results suggest that it may be beneficial to perform sub-group analyses of the individual VA and DoD populations, in addition to the combined population analysis, in order to better characterize disease trends. The benefits of joint VA/DoD biosurveillance were also suggested based on comparisons between the combined population with the VA-only and DoD-only component sub-analysis performed at the NC-VAMC/Lovell FHCC. In situations where health threats exist, accurate and readily available disease surveillance data is critically important, as it not only improves the execution of emergency management procedures, but also the appropriate and timely diversion of resources. Furthermore, early detection of disease trends could alert facilities with large numbers of active duty personnel and recruits (such as Great Lakes Naval Training Center) to take appropriate public health actions to avoid compromise of operational readiness. This analysis provided additional impetus for the submission of a Joint Incentive Fund (JIF) proposal in support of the development of a combined VA/DoD ESSENCE Biosurveillance System. Both agencies annually contribute funds to the DoD-VA Health Care Sharing Incentive Fund which provides seed money through the JIF project process for creative sharing initiatives designed to facilitate coordination, use, or exchange of health care resources with the goal of improving health care provided to beneficiaries of both departments.

### Future Steps

In August 2009, the VA/DoD National Biosurveillance JIF project proposal was approved and $4.8 million was appropriated for the initial phases of the project. We believe this project supports exploration in innovative surveillance methods and performance measures which have long been advocated [34-38]. The overarching goal of this effort is to position two well established biosurveillance programs to collaborate on the analysis of health surveillance data, improve information and knowledge sharing between our agencies and enhance biosurveillance capabilities. Coding evaluations and system development work have been performed independently by each agency over the years [32,39-42]. Because each system has been functioning independently and both versions have made unique upgrades and enhancements over the years, the best features of each system can be incorporated into the joint system, which would ultimately provide more functionality than either system independently. For example, DoD ESSENCE has successfully incorporated patient disposition and some vital sign data into their system which would be a valuable addition to current VA functionality. VA ESSENCE is upgrading graphing and mapping functionality which would be an improvement over what the DoD system currently has available. A bidirectional VA/DoD surveillance system advances the goals and priorities outlined in the Homeland Security Presidential Directive-21 and the National Biosurveillance Strategy for Human Health 2008-2013. These documents have prioritized improving existing biosurveillance infrastructure, fostering collaboration between public health professionals responsible for biosurveillance within different agencies and coordination of health-related information sharing across all levels of government to improve the effectiveness of disease monitoring and response.

The system created through the JIF project will contain data for all VA and DoD facilities. This pilot analysis represented a unique situation where a VA and DoD facility physically merged which provided the opportunity, in advance of the launch of the JIF project, to evaluate data from VA and DoD populations in a single surveillance application (the VA ESSENCE system). However, the joint system will differ in that VA and DoD facilities will remain distinct entities although there will be opportunities with this system to look at surveillance data for cities and regions where both VA and DoD facilities exist in close proximity to each other or where sharing agreements exist. In a way, the combined system will more closely mimic ESSENCE systems that have been established in state and local health departments, which incorporate data from distinct healthcare systems/hospitals from the civilian sector in close geographic proximity. In the future, there may also be opportunities for sharing ESSENCE data with state or local ESSENCE systems for even greater situational awareness in communities containing both Federal and civilian healthcare facilities.

### Limitations

This analysis was limited to a single facility and was a retrospective review of surveillance data. Although a number of VA facilities have DoD patient sharing agreements, North Chicago was chosen for this initial demonstration project due to the large volume of DoD patients seen at the facility and the proximity of two other VA Medical Centers in the same metropolitan area for comparison. In the future, real-time analysis of surveillance data across multiple facilities during actual health events will be important in determining the usefulness of a combined population for situation awareness. The JIF joint surveillance project will be a much larger data integration effort, which will provide additional information to analyze the utility of integrated VA and DoD surveillance data for event detection and situational awareness. Secondly, since the surveillance system relies solely on ICD-9 diagnosis codes, coding errors may impact the quality of the data. Thirdly, there were relatively few large-scale public health events identified for this time period through the Chicago Health Department online archives for inclusion in this analysis. A review of county, state or other resources might be helpful in identifying additional historical events that could be analyzed in the future. Finally, since we did not review patients' clinical records we cannot determine whether cases with GI syndrome codes or heat injury codes were related to the specific events identified during the time periods in question.

## Conclusions

Analysis of biosurveillance data in this joint VA/DoD facility suggested the potential for improved ILI trend monitoring and temporal alerting in a combined biosurveillance application. The combined population in this first Federal Health Care facility represents a larger statistical sample of the total population (including pediatric) in the region, which may result in improved recognition of disease events and ILI trends compared to the VA population alone. Joint VA/DoD biosurveillance may be beneficial in other areas where VA and DoD facilities are in close proximity or where facility sharing agreements exist.

## Competing interests

The authors declare that they have no competing interests.

## Authors' contributions

CAL formulated the study design, performed data queries, researched historical events and comparative data, analyzed the results and drafted the manuscript. GO and MH participated in the study design, interpretation of data and helped draft the manuscript. KC assisted in acquisition of DoD data, interpretation of data and revision of the manuscript. FM assisted in acquisition of VA data, interpretation of data and revision of the manuscript. JL and RW assisted in acquisition of ESSENCE data and revision of the manuscript. All authors read and approved the final manuscript.

## Pre-publication history

The pre-publication history for this paper can be accessed here:

http://www.biomedcentral.com/1472-6947/11/56/prepub
